# TUMOR MARKERS EXPRESSION LEVELS IN GASTRIC CANCER PATIENT’S PERIPHERAL BLOOD BY RT-PCR ASSESSMENT

**DOI:** 10.1590/0102-672020230071e1789

**Published:** 2024-02-05

**Authors:** Gabriel da Silva KAWAKAMI, Marina Alessandra PEREIRA, Márcia Saldanha KUBRUSLY, Alexis Germán Murillo CARRASCO, Marcus Fernando Kodama Pertille RAMOS, Ulysses RIBEIRO

**Affiliations:** 1 Universidade de São Paulo, Instituto do Câncer, Hospital das Clínicas, Faculty of Medicine, Department of Gastroenterology, São Paulo (SP), Brazil.

**Keywords:** Stomach Neoplasms, Gastrectomy, Tumor Biomarkers, Neoplasm Metastasis, Reverse Transcriptase Polymerase Chain Reaction, Neoplasias Gástricas, Gastrectomia, Biomarcadores Tumorais, Metástase Neoplásica, Reação em Cadeia da Polimerase Via Transcriptase Reversa

## Abstract

**BACKGROUND:**

Hematological recurrence is the second most frequent cause of failure in the treatment of gastric cancer. The detection of circulating tumor markers in peripheral blood by quantitative reverse transcriptase polymerase chain reaction (qRT-PCR) method may be a useful tool to predict recurrence and determine the patient’s prognosis. However, no consensus has been reached regarding the association between the tumor markers level in peripheral blood and its impact on patient survival.

**AIMS:**

To evaluate the expression of the circulating tumor markers CK20 and MUC1 in peripheral blood samples from patients with gastric cancer by qRT-PCR, and to verify the association of their expression levels with clinicopathological characteristics and survival.

**METHODS:**

A total of 31 patients with gastric adenocarcinoma were prospectively included in this study. CK20 and MUC1 expression levels were analyzed from peripheral blood by the qRT-PCR technique.

**RESULTS:**

There was no statistically significant (p>0.05) association between CK20 expression levels and clinical, pathological, and surgical features. Higher MUC1 expression levels were associated with female patients (p=0.01). There was a correlation between both gene levels (R=0.81, p<0.001), and CK20 level and tumor size (R=0.39, p=0.034).

**CONCLUSIONS:**

CK20 and MUC1 expression levels could be assessed by qRT-PCR from total peripheral blood samples of patients with gastric cancer. CK20 levels were correlated to MUC1 levels as well as to tumor size. There was no difference in disease-free survival and overall survival regarding both genetic markers expression in this series.

## RESUMO

**RACIONAL:**

A recorrência hematológica é a segunda causa mais frequente de falha no tratamento do câncer gástrico. A detecção de marcadores tumorais circulantes no sangue periférico, pelo método de reação em cadeia da polimerase de transcrição reversa quantitativa (qRT-PCR) pode ser uma ferramenta útil para prever a recorrência e determinar o prognóstico do paciente. No entanto, ainda não foi alcançado consenso em relação à associação entre o nível de marcadores tumorais circulantes no sangue periférico e seu impacto na sobrevida do paciente.

**OBJETIVOS:**

Avaliar a expressão de CK20 e MUC1 em amostras de sangue periférico de pacientes com câncer gástrico por meio de qRT-PCR e verificar a associação dos níveis de expressão com características clinicopatológicas e sobrevida.

**MÉTODOS:**

Trinta e um pacientes com adenocarcinoma gástrico foram incluídos, prospectivamente. Os níveis de expressão de CK20 e MUC1 foram analisados a partir de sangue periférico por meio de qRT-PCR.

**RESULTADOS:**

Não houve associação estatisticamente significativa (p>0,05) entre os níveis de expressão de CK20 com características clínicas, patológicas e cirúrgicas. Níveis mais elevados de expressão de MUC1 estavam associados a pacientes do sexo feminino (p=0,01). Houve correlação entre os níveis de ambos os genes (R=0,81, p<0,001), nível de CK20 e tamanho do tumor (R=0,39, p=0,034).

**CONCLUSÕES:**

Os níveis de CK20 e MUC1 podem ser avaliados por qRT-PCR a partir de amostras de sangue periférico total de pacientes com câncer gástrico, os níveis de CK20 estavam correlacionados com os de MUC1, assim como tamanho do tumor. Não houve diferença de sobrevida global ou livre de doença em relação à expressão de ambos marcadores genéticos nesta série.

## INTRODUCTION

Gastric cancer (GC) is the 5th most common cancer worldwide^6^, and adenocarcinoma is the most common histological type^1,26^ usually diagnosed at an advanced stage that requires gastrectomy and lymphadenectomy^25^.

Since hematological recurrence is the second most frequent cause of failure after potentially curative treatment of GC^5^, the detection of circulating tumor markers (CTM) in peripheral blood, by quantitative reverse transcriptase polymerase chain reaction (qRT-PCR) method may be a useful tool to predict recurrence and determine the patient’s prognosis^7,9,36^. However, no consensus has been reached regarding the association between the presence of CTM in peripheral blood and its impact on patient survival^9,10,14,18,27,32,35^.

Detection of CTM can be accomplished by immunohistochemistry (IHQ) or RT-PCR. The former is based on the use of tumor cells epithelial markers which can be identified by specific monoclonal antibodies. On the other hand, for RT-PCR, tumor protein codifying mRNA is used to synthesize complementary DNA, which is amplified through multiple replications in order to allow the detection of CTM^2,7,12,18,22,30^.

The qRT-PCR method is more sensitive and faster, enabling it to detect a greater number of CTM than IHQ, once the latter is capable of just analyzing a few cuts up to 6 μm thick that represents less than 1% of the lymph node total volume^2,14,19,20,23^. Some researchers indicate that the exam’s high sensitivity may reveal only the presence of tumoral DNA and not necessarily the presence of viable tumoral cells, so it may present false positive results^23,38^. However, the use of multiple markers for RT-PCR may increase the exam’s sensitivity and avoid false positives, providing better detection of tumor markers that would not be recognized through techniques such as IHQ^2,21^.

Cytokeratins (CK) are cytoskeletal compounds that aim to fix the nucleus and maintain the cell’s morphology. There are 20 subtypes of CK and its expression depends on the epithelial cell type and its differentiation degree. Thus, CK expression is detected in gastrointestinal carcinomas^23,33^. The use of IHQ for the detection is suitable^15,16,23^, thereby 27.0% positive cases for CK20 were reported using this method^33^. Peripheral blood sample RT-PCR analysis found CK20 positivity in 37.1%^7^ and 27.3%^10^ of GC patients, while the result using lymph nodes analyzed by the same technique was 23.2%^14^.

MUC1 is one of 14 genes responsible for mucin production which composes the stomach protector mucus layer^24^. The detection of MUC1 through IHQ has different results depending on the antibody used, but all present expression values up to 50.0%^24,28^. By searching for tumoral cells in peripheral blood samples using RT-PCR, Uen et al. registered 71.2% MUC1 positive cases in GC patients^35^.

Thus, the present study aimed to evaluate the expression of the circulating tumor markers CK20 and MUC1 in peripheral blood samples from GC patients, who underwent gastrectomy, and to verify the association of their expression levels with clinicopathological characteristics and survival.

## METHODS

### Patients

Thirty-one patients diagnosed with GC who underwent potentially curative gastrectomy between July 2014 and January 2016 in our center were prospectively included in the study.

Inclusion criteria were diagnosis of gastric adenocarcinoma confirmed by histological examination, no neoadjuvant treatment, and absence of metastatic disease at the time of diagnosis. Blood samples (15 mL) were collected at enrollment, during outpatient consultation, or on admission prior to surgery. The samples were processed and stored in the freezer at -80°C until the moment of use.

Clinical, surgical, and pathological data were collected from electronic records, including: age, sex, body mass index (BMI) (kg/m^2^), adjuvant chemotherapy, American Society of Anesthesiologists classification (ASA), type of gastrectomy, tumor location, Lauren’s histologic type, degree of differentiation, number of dissected lymph nodes, lymphatic, venous and perineural invasions, tumor size (cm), and pTNM staging. Tumor stage was defined according to the 8th edition of the TNM classification.

Patients were followed-up according to standard protocol every three months during the first year, every six months during the second and third years, and once a year thereafter, in the outpatient clinical visits.

Overall survival was calculated from the time of surgery until death or last observation. Disease-free survival comprised the time between surgery until recurrence, death, or last observation.

The study was approved by the institutional review board of our hospital and registered on plataformabrasil.saude.gov.br, CAEE: 19912713.2.0000.0065.

### RNA extraction from peripheral blood

Total RNA was extracted from blood samples using TRIzol® kit (guanidinium isothiocyanate) following the manufacturer’s recommendation. The concentration was determined by a NanoDrop tND-1000 spectrophotometer (NanoDrop Technologies, Inc. Wilmington, USA) and a 260/280 nm absorbance ratio was used to determine the RNA purity (values equal to 1.8 were considered ideal for use). The RNA quality was assessed by gel electrophoresis (28S and 18S bands visualization), then samples were stored at -80°C freezers until being used.

### cDNA conversion

The cDNA was synthesized using the High-Capacity RNA-to-cDNA Kit (Applied Biosystems, USA) according to the manufacturer’s instructions. Briefly, reactions were incubated for 60 min at 35°C and 5 min at 95°C in the GeneAmp 2400 Thermocycler (Applied Biosystems). The resulting cDNA was stored at -20°C until being used for qRT-PCR reactions.

### qRT-PCR

The genetic expression analyses were performed by qRT-PCR in the StepOnePlus™ Thermocycler (Applied Biosystems, Life Technologies, Foster City, EUA) using TaqMan® Gene Expression Assays (Applied Biosystems). CK20 (Hs00300643_m1), MUC1 (Hs00159357_m1), and GAPDH (Hs02786624_g1) assays were obtained from Thermo Fisher Scientific.

All qRT-PCR amplifications were performed in a total volume of 20.0 μL, each reaction containing 10.0 μL of TaqMan® Gene Expression Master Mix 2X, 1.0 μL of Taqman® Gene Expression Assay 20X, 5.0 μL of RNase-DNase free water, and 4.0 μL cDNA (1 to 100 ng). Cycling parameters were 2 min at 50°C, 10 min at 95°C, 40 cycles of 15 seconds at 95°C, and 1 min at 60°C.

Relative quantification values (RNA expression-related levels) were calculated by Delta-Delta Comparative Threshold Method (DeltaDeltaCt) with internal control to correct differences. The threshold-cycle value (Ct) for each target gene was normalized through GAPDH gene Ct mean value subtraction (DeltaCt = target Ct - internal control Ct) (Livak & Schmittgen, 2001).

### Statistical analysis

Gene expression and clinical quantitative features were compared with qualitative information according to the data distribution evaluated by the Kolmogorov-Smirnov test. If quantitative data followed a normal distribution, we used the t-test to compare the two groups. Otherwise, the Mann-Whitney (unpaired Wilcoxon) test was applied. For comparison with more than two categories, we applied the Kruskal-Wallis test.

For correlation evaluations, we applied Pearson’s chi-square test method between quantitative variables, while the Kaplan-Meier method was used for evaluating prognosis features in disease-free survival and overall survival. It was considered Pearson’s coefficient to determine its strength as weak (<0.4), moderate (0.4–0.6), or strong (>0.6).

For performing these analyses and plotting, survival, boxplots, dot plots, and scatter plots, we used the R software v.4.3.0.

## RESULTS

### Study cohort

Among the 31 patients included, 58.4% were male and the mean age was 59.9 years (ranging 22.8–81.5). Most patients underwent subtotal gastrectomy (77.4%), and D2 lymphadenectomy was performed in 93.5% of cases.

Intestinal type was the most common histologically, and most patients were staged as pTNM stage III (38.7%). Sixteen (51.6%) patients underwent adjuvant chemotherapy. Clinical, surgical, and pathological characteristics of patients are shown in Table 1.

**Table 1:** Clinical, surgical and pathological characteristics of 31 patients with gastric cancer included in the study.

Variables	n=31	%
Sex		
Female	14	45.2
Male	17	54.8
Age (years)		
Mean (SD)	59.9 (14.3)	
Min–max	22.8–81.5	
BMI (kg/m²)		
Mean (SD)	23.9 (4.36)	
American Society of Anesthesiologists		
II	25	80.6
III	6	19.4
Tumor location		
Distal	21	67.7
Medial	8	25.8
Proximal	2	6.5
Type of gastrectomy		
Subtotal	24	77.4
Total	7	22.6
Type of lymphadenectomy		
D1	2	6.5
D2	29	93.5
Tumor size (cm)		
Mean (SD)	5.52 (3.75)	
Lauren type		
Intestinal	17	54.8
Diffuse/mixed	14	45.2
Tumor differentiation		
Well/moderately differentiated	14	45.2
Poorly differentiated	17	54.8
Nº of resected lymph nodes		
Mean (SD)	37.35 (8.89)	
Lymphatic invasion		
No	20	64.5
Yes	11	35.5
Venous invasion		
No	22	71.0
Yes	9	29.0
Perineural invasion		
No	16	51.6
Yes	15	48.4
pT		
T1	9	29.0
T2	4	12.9
T3	13	41.9
T4	5	16.1
pN		
pN0	12	38.7
pN+	19	61.3
pTNM		
I	11	35.5
II	7	22.6
III	12	38.7
IV	1	3.2

BMI: body mass index; SD: standard deviation; TNM: tumor, node, metastasis staging system; Min: minimum; Max: maximum.

### CK20 results

CK20 mean level of detection was 13.11 (-DeltaCt) among all 31 blood samples, with a standard deviation (SD) of ±2.32. The median level was 12.87 with an interquartile range of 11.13 to 15.15. The minimum value detected was 9.23, while the maximum was 18.32 (Table 2).

**Table 2:** Comparison between CK20 expression and clinicopathological characteristics of thirty-one patients with gastric cancer included in the study.

Variables	CK20-DeltaCt Median (IQR)	p-value
Sex		
Female	11.41 (12.85–11.02)	0.38
Male	14.89 (15.80–12.59)	
Tumor location		
Distal	11.43 (12.99–10.87)	0.55
Other	15.62 (16.63–14.96)	
Lauren type		
Intestinal	14.89 (15.80–12.59)	0.17
Diffuse/mixed	11.41 (12.85–11.02)	
Lymphatic invasion		
No	11.41 (12.90–10.85)	0.87
Yes	16.45 (15.44–15.02)	
Venous invasion		
No	11.72 (13.14–10.90)	0.68
Yes	15.80 (16.81–14.89)	
Perineural invasion		
No	11.41 (12.58–10.93)	0.25
Yes	15.95 (15.15–13.22)	
pT		
T1-T2	11.39 (12.99–10.98)	0.06
T3-T4	14.65 (15.71–12.22)	
pN		
pN0	11.7 (13.04–10.93)	0.27
pN+	14.40 (15.62–11.76)	
pTNM		
I-II	11.41 (12.76–10.83)	0.09
III-IV	15.27 (16.10–14.40)	

CK: Cytokeratins; Ct: threshold-cycle value; DeltaCt: target Ct – internal control Ct; IQR: interquartile range; TNM: tumor, node, metastasis staging system.

**Figure 1:**
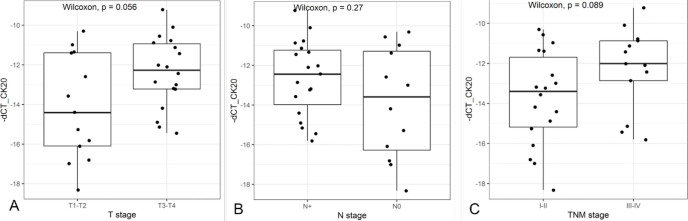
Comparison between CK20 expression and clinicopathological features: (A) T stage; (B) N stage; (C) TNM stage.

Even though it did not reach a significant value, there was a trend toward a difference related to the T stage in which high marker expressions were associated with larger tumors. However, there were no statistically significant associations between CK20 expression levels and clinical, pathological, and surgical characteristics. The boxplots related to T, N, and TNM stages are shown in Figure 1.

CK: cytokeratins.

### MUC1 results

MUC1 mean level of detection was 10.05 (-DeltaCt), with SD±2.27. The median level was 9.47 with an interquartile range of 8.15 to 12.03. The minimum value detected was 7.16, while the maximum was 14.78 (Table 3).

**Table 3:** Comparison between MUC1 expression and clinicopathological characteristics of 31 patients with gastric cancer included in the study.

Variables	MUC1-DeltaCt Median (IQR)	p-value
Sex		
Female	8.15 (9.04–7.58)	0.01
Male	11.82 (12.85–9.87)	
Tumor location		
Distal	8.76 (9.47–7.72)	0.20
Other	12.57 (13.83–12.07)	
Lauren type		
Intestinal	11.82 (12.85–9.87)	0.06
Diffuse / mixed	8.15 (9.04–7.58)	
Lymphatic invasion		
No	8.69 (9.22 –7.72)	0.89
Yes	12.30 (13.79–11.92)	
Venous invasion		
No	8.85 (9.53–7.80)	0.72
Yes	12.85 (13.88–12.12)	
Perineural invasion		
No	8.38 (9.22–7.67)	0.22
Yes	13.27 (12.03–10.42)	
pT		
T1-T2	8.14 (9.07–7.53)	0.09
T3-T4	11.63 (12.71–9.32)	
pN		
pN0	7.93 (9.07–7.52)	0.92
pN+	11.45 (12.58–9.11)	
pTNM		
I-II	8.38 (9.12–7.72)	0.44
III-IV	12.12 (13.70–11.45)	

**Figure 2:**
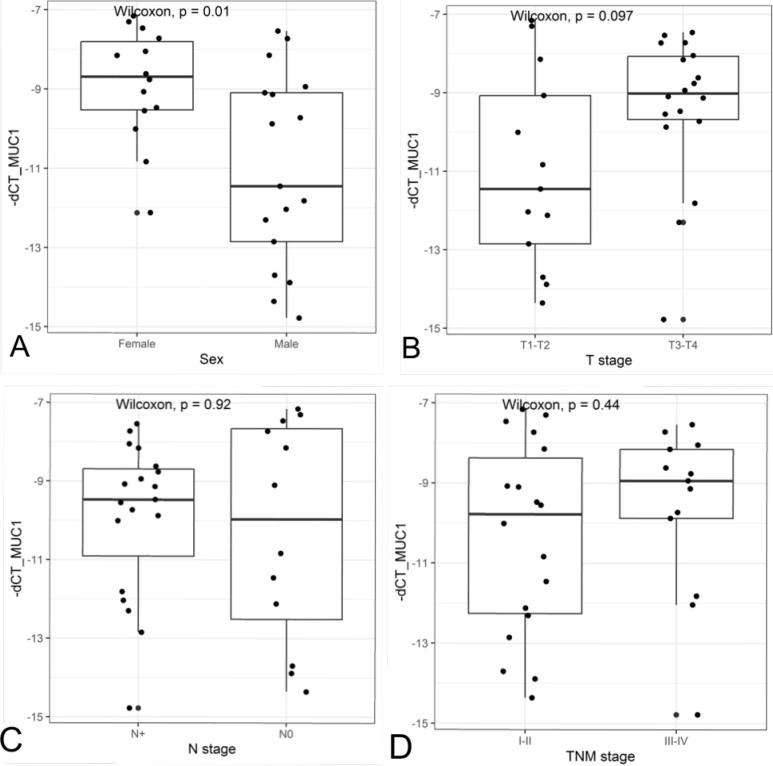
Comparison between MUC1 expression and clinicopathological features: (A) sex; (B) T stage; (C) N stage; (D) TNM stage.

TNM: tumor, node, metastasis staging system; MUC: mucin production gen; Ct: threshold-cycle value.

MUC: mucin production gen.; Ct: threshold-cycle value; DeltaCt: target Ct – internal control Ct; IQR: interquartile range; TNM: tumor, node, metastasis staging system;

There was an association between MUC1 expression levels and sex (p=0.01). Other comparisons were not statistically significant. The boxplots related to sex and pT, pN, and pTNM stages are shown in Figure 2.

### Correlation results

**Figure 3:**
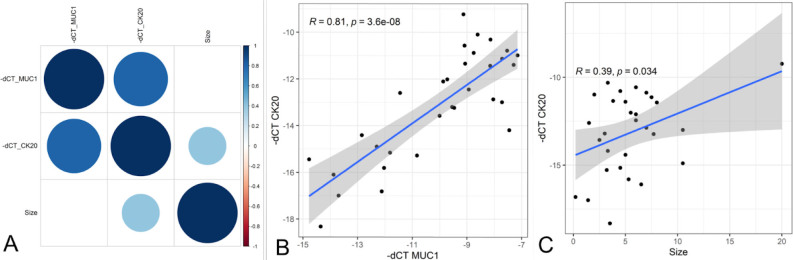
Correlation results between gene expression and tumor size. (A) Correlation map of quantitative variables; (B) Correlation between CK20 and MUC1 expression levels; (C) Correlation between CK20 expression levels and tumor size.

CK: cytokeratins; MUC: mucin production gen; Ct: threshold-cycle value.

Correlation analyses between MUC1 and CK20 expression with quantitative variables are demonstrated in Figure 3.

There was a positive correlation between both gene expression levels (R=0.81, p<0.001), and between CK20 expression levels and tumor size (R=0.39, p=0.034).

### Overall survival and disease-free survival

In a mean follow-up of 38.5 (±23.6) months, 11 (35.5%) patients had disease recurrence and 12 (38.7%) died.

For survival analyses, patients were divided into low- and high-level expression groups based on median values (CK20 median=-12.87; MUC1 median=-9.47).

**Figure 4:**
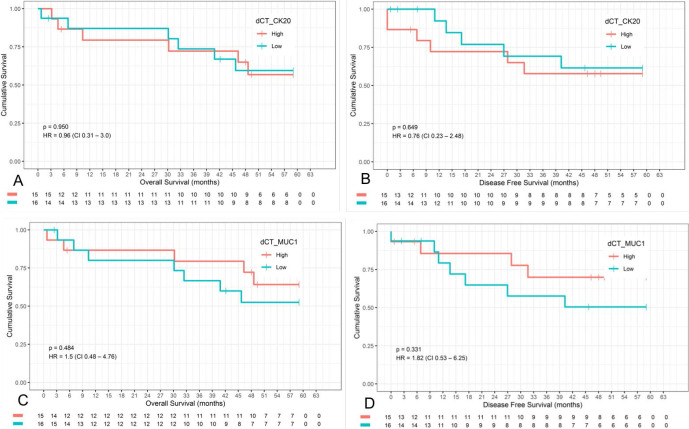
Overall survival and disease-free survival by median value: (A) OS according to CK20 expression levels; (B) DFS according to CK20 expression levels; (C) OS according to MUC1 expression levels; (D) DFS according to MUC1 expression levels.

OS: overall survival; DFS: disease-free survival; CK: cytokeratins; MUC: mucin production gen; Ct: threshold-cycle value.

There was no difference in disease-free survival and overall survival according to CK20 and MUC1 expression (Figure 4).

## DISCUSSION

The current study aimed to evaluate the expression of circulating tumor markers CK20 and MUC1 in peripheral blood samples from GC patients by qRT-PCR. We found a positive correlation between both gene expression levels, a rising CK20 detection pattern for larger tumors, and also a higher MUC1 expression related to female patients. No other statistically significant results were found between the analyzed genes and clinicopathological features, as well as disease-free survival and overall survival.

The qRT-PCR method has been increasingly used to detect CTM in various tissues, such as peripheral blood, lymph nodes, and bone marrow due to a higher sensitivity compared to immunohistochemistry^2,7,12,14,18,22,30^. Furthermore, the use of multiple markers in qRT-PCR technique may provide a sensitivity increase and false-positive rates decrease, allowing better CTM detection^2,7,21^.

CTM detection has been associated with patient’s worst prognosis in several studies^4,7,9,11,13,17,21,23,24,34-37^. The use of qRT-PCR technique sheds light on the early detection of recurrence even though no consensus about its significance and impact to patient’s survival has been reached until the present moment^9,10,14,18,27,32,35^.

For this study purpose, total blood without any type of centrifugation nor cellular enrichment analysis was chosen due to higher financial viability in the real context of the technique use for genetic markers research. Thus, peripheral blood samples were taken before surgical procedures, upon signed free and informed consent. Additionally, the Cancer Institute of the State of São Paulo (ICESP, acronym in Portuguese) biobank standard sample collection protocol was adopted in order to avoid contamination by epithelial cells.

A quantitative RT-PCR approach was adopted to analyze the marker’s expression, in contrast to the majority of existing studies in which a cutoff value representing the presence or absence of micrometastasis is usually used^7,8,10,11,13,29,31,34,35^. The reason behind this choice is that total blood RNA samples would not allow to attribute the obtained expression value to metastasis occurrence itself, since no neoplastic cellular enrichment method was used. Thus, the current study correlates expression levels of genetic markers with clinical and pathological parameters in order to assess the utility of total blood quantitative analysis use for prognosis monitoring, survival, and GC patient staging.

An increased expression level for both genes could be observed during quantitative variables analysis. CK20 expression rates were associated with higher MUC1 rates and vice versa. Also, higher CK20 expression correlated with larger tumor size. These findings match with the existing literature about the increase in genetic expression values in GC patients, in relation to CK20^7,10,11,17^ as well as to MUC1^29,34,35^.

Besides that, higher MUC1 expression was associated with female patients (p=0.01) but this association does not have a clear explanation in the literature.

Even though not reaching the stipulated cutoff value for being considered statistically significant, other associations (N, T, and TNM stages) related to CK20, as well as MUC1 are also pointed out. All of them demonstrated an expected behavior of higher genetic expression for more aggressive tumor types^7,10,11,17,29,34,35^, which includes: lymph node involvement, larger tumor, higher TNM stage, and diffuse type of tumor. Thus, the low number of included patients may be a limiting factor for obtaining greater statistical relevance in these comparisons.

There is great heterogeneity in the literature in terms of CTM analysis methodology, which hampers the comparison between this and other studies. It can be cited the differences related to sample types (lymph node or peripheral blood), handling of sample technique (total blood or neoplastic cellular enrichment methods), employed analysis type (immunohistochemistry or RT-PCR), the establishment of a cutoff value from which it can be considered positive for micrometastasis, and positive and negative control groups employment^3^. Thereby, each marker positivity percentage would not be approached, since this perspective does not suit the current study methodology.

Therefore, despite inexistent consensus concerning the use of the RT-PCR technique and the use of CK20 and MUC1 markers for CTM detection and prognosis, staging and recurrence assessment, published studies obtained statistically significant results for clinical and pathological analysis, as well as a correlation between higher gene expression and worse prognostic tumors^4,7,17,23,27,33-35^. Thus, our results correspond to those existing in the literature, since higher genetic expression was associated with larger tumors.

In the analysis of overall survival and disease-free survival curves related to marker’s expression, it was not found to have a statistically significant association with gene expression. Beyond that, only the survival related to CK20 expression showed an expected behavior of worse outcome for higher marker expression levels as it has been reported in the literature^4,11,17,24,35,37^. This can probably be interpreted as a random finding once the difference in the number of patients between groups is considerably significant.

This research has some limitations. First, the number of patients is small; second, we just evaluated two circulating tumor markers, CK20 and MUC1; and third, we examined total peripheral blood. By increasing the number of patients and molecular markers, and blood enrichment, we could improve the outcome of the research.

To sum up, the use of genetic markers in the peripheral blood sample analysis by qRT-PCR to assess survival, prognosis, and staging of GC patients is promising and deserves to be better explored with a larger number of patients and more genetic markers in order to improve the exam sensitivity. Besides that, the use of peripheral blood sample purification techniques could be useful to assess whether the increase of marker expression is truly due to a larger amount of CTM existing in the sample.

Therefore, the results obtained in this research do match those existing in the literature in terms of the association between higher marker expression levels and advanced staging tumors.

## CONCLUSIONS

CK20 and MUC1 expression levels could be assessed by qRT-PCR from total peripheral blood samples of GC patients. CK20 levels were correlated to MUC1 levels as well as to tumor size. There was no difference in disease-free survival and overall survival according to both genetic markers in this series.
